# Technical viability of the YF MAC-HD ELISA kit for use in yellow fever-endemic regions

**DOI:** 10.1371/journal.pntd.0009417

**Published:** 2021-06-04

**Authors:** Christin H. Goodman, Maurice Demanou, Mick Mulders, Jairo Mendez-Rico, Alison Jane Basile

**Affiliations:** 1 Centers for Disease Control and Prevention, Fort Collins, Colorado, United States of America; 2 World Health Organization Regional Office for Africa, Ouagadougou, Burkina Faso; 3 World Health Organization Department of Immunizations, Vaccines, and Biologicals, Geneva, Switzerland; 4 Pan-American Health Organization, Washington District of Columbia, United States of America; KU Leuven, Rega Institute, BELGIUM

## Abstract

Yellow fever (YF), an arboviral disease, affects an estimated 200,000 people and causes 30,000 deaths per year and recently has caused major epidemics in Africa and South America. Timely and accurate diagnosis of YF is critical for managing outbreaks and implementing vaccination campaigns. A YF immunoglobulin M (IgM) antibody-capture (MAC) enzyme-linked immunosorbent assay (ELISA) kit, the YF MAC-HD, was successfully introduced starting in 2018 to laboratories in Africa and South America. The YF MAC-HD kit can be performed in 3.5 hours, test up to 24 samples, and includes all reagents necessary to perform the test, except for water used to dilute wash buffer. In 2018 and 2019, a total of 56 laboratory personnel from 39 countries in Africa and South America were trained to use the kit during workshops, followed by take-home YF IgM proficiency testing (PT) exercises. Participants received either a 10- or 20-sample YF PT panel and performed testing using the YF MAC-HD kit. All countries obtained 90% or higher correct results. These results verified the technical viability and transferability of YF MAC-HD kit use for laboratories in YF-endemic countries.

## Author summary

Yellow fever is a vaccine-preventable disease transmitted by mosquitoes that annually affects an estimated 200,000 people and causes 30,000 deaths. Being able to quickly and accurately identify people infected with yellow fever virus by laboratory confirmation is critical for managing outbreaks and vaccination campaigns. A test developed by the Centers for Disease Control and Prevention can identify yellow fever antibodies in a person’s blood within approximately four hours. In order to assure this test could be used in laboratories located in regions with yellow fever transmission, workshops were held to train laboratorians from Africa and South America on how to use the test. Laboratorians were then given a panel of samples and performed the test in their own laboratories. Of the 39 countries that performed testing, all countries scored 90% or higher concordant results, demonstrating the successful transfer of this yellow fever antibody detection test.

## Introduction

Yellow fever (YF) is an arboviral disease endemic in tropical and subtropical areas of Africa and the Americas. It is estimated to cause 200,000 cases and 30,000 deaths annually, with 90% occurring in Africa [1,2]. The causative agent of YF, yellow fever virus (YFV), is a single-stranded RNA virus from the genus *Flavivirus*, family *Flaviviridae*, and is primarily spread via *Aedes spp*. and *Haemagogus spp*. mosquitoes [3]. Most individuals who become infected with YFV are either asymptomatic or develop mild, non-specific illness that may consist of fever, headache, body aches, fatigue, nausea, or vomiting [1,3,4]. Approximately 5–26% of symptomatic individuals, however, develop more severe YF disease, consisting of high fever, the typical jaundice, bleeding, shock, and organ failure; of those who develop severe YF disease, 30–60% will die [4,5].

YF epidemics have occurred in recent years in both Africa and the Americas. In South America in 2016 to June 2020, multiple YF sylvatic epidemics and epizootics occurred in Brazil, notably near the large urban areas of São Paulo and Rio de Janeiro [6,7,8]. These Brazilian YF epidemics led to at least 2,278 confirmed human cases and 777 deaths [6,9]. On the African continent, YF outbreaks were reported in Angola and the Democratic Republic of the Congo in 2015 and 2016, leading to more than 7,334 suspected cases, 962 of which had been laboratory-confirmed, and 393 registered deaths [10]. From January to December 2019, Nigeria reported 4,288 suspected yellow fever cases, 227 which had been laboratory-confirmed, and 231 deaths [11,12]. Despite an effective and safe vaccine that prevents YF disease and induces likely lifelong, protective immunity, many people living in or near at-risk territory remain unvaccinated [13].

In 2017, the World Health Organization (WHO) launched the Global Strategy to Eliminate Yellow Fever Epidemics (EYE) 2017–2026, a global coalition of countries and partners to help combat the increased risk of YF epidemics [13]. One of the strategic objectives of the EYE Strategy is to contain outbreaks rapidly [13], the success of which hinges, in part, on high quality YF diagnostic testing. The need for rapid and accurate YF diagnostic tests is of paramount importance because in many people early symptoms of YF are clinically indistinguishable from those caused by many other acute infections. However, there is currently a lack of validated commercially available serological and molecular assays [14]. Laboratories use non-standardized in-house YF assays and countries often rely on a small number of reference laboratories to perform YF testing [4,15]. The availability of validated, standardized YF assays would greatly aid in filling these critical diagnostic gaps [14].

Immunoglobulin M (IgM) antibody testing for YF is one of the primary diagnostic methods used because it is the initial humoral isotype response generated following an infection [15]. Due to cross-reactivity with other flaviviruses [16], YF IgM testing is used as a primary screening method and YF IgM positive results require confirmatory neutralization testing. In 2015, a standardized YF IgM antibody capture (MAC) enzyme-linked immunosorbent assay (ELISA) kit (YF MAC-HD) was developed by the Centers for Disease Control and Prevention (CDC) that could be completed in approximately 3.5 hours [15]. Each kit can be used to test up to 24 serum samples, and all the required reagents, except for water in which to dilute the wash buffer, are included in the kit [15]. Development of the YF MAC-HD kit was based on the CDC YF MAC-ELISA, an in-house test that uses fourteen individual reagents and commercially sourced components and requires an overnight antigen incubation step [17]. However, obtaining all these individual reagents and components often poses challenges for countries that are resource-limited, and stock-outs are common. Additionally, critical reagents such as antigen and conjugate need to be titrated by the testing laboratories, which subsequently may be leading to standardization challenges. Implementation of the YF MAC-HD kit would help streamline and standardize testing across laboratories, mitigate reagent issues, eliminate the need for titration and validation of individual reagents, and shorten the testing time to 3.5 hours. If the kit proves viable for use in high risk YF-endemic regions, it could lead to more reliable YF surveillance and outbreak response.

YF IgM proficiency testing (PT) panels were developed and used to assess the effectiveness of trainings that introduced the YF MAC-HD kit and to determine the extent to which the YF MAC-HD kit technology is transferable to, and technically viable in, laboratories of YF-affected regions. This report describes how performance and technical viability of the kit in YF-endemic settings was evaluated.

## Methods

### Ethics statement

Residual human specimens were used according to the Centers for Disease Control and Prevention Institutional Review Board protocol 6773. Formal consent was not obtained from the training participants due to anonymization of results of participants and countries.

The YF MAC-HD kit was produced using Good Documentation Practices (GDocP) by the Bio-pharmaceutical Manufacturing & Academic Resource Center (BioMARC), a non-profit biologics contract development and manufacturing organization owned and operated by Colorado State University (CSU). The kit was first introduced to laboratory experts during a YF MAC-HD training workshop held in Fort Collins, Colorado, USA in May 2018. Five laboratory experts from five countries in Africa and five laboratory experts from four countries in South America at high or moderate risk of YF attended this week-long workshop where they trained and performed testing using the kit. Test results were calculated both manually and by using an Excel workbook with embedded formulae to automate the calculations.

At the end of the workshop, 10 kits were donated and shipped to each country for use in proficiency testing, staff training, and testing of archived samples. A 20-sample YF PT panel accompanied the kits, where the panel consisted of 11 serum samples of varying high (5), medium (4), and low (2) YF IgM positivity and 9 negative IgM serum samples. The YF IgM positive samples were previously confirmed via neutralization assays and selected based on their respective P/N (defined as the mean optical density (OD) of the sample reacted on YF antigen divided by the mean OD of the negative control reacted on YF antigen) and NBR (nonspecific background reaction; defined as the mean OD of the sample reacted on YF antigen divided by the mean OD of the sample reacted on normal antigen) values. When tested at the CDC, high, medium, and low YF IgM positive samples had approximate P/N values of >11, 6–8, and 3–4, respectively, and all had NBR values of ≥1.5. The negative IgM samples had P/N values of <1.5. Each vial contained 25ul of serum that was heat-inactivated at 56°C for 30 minutes to help reduce sample infectivity. Reference results were obtained at the CDC using the YF MAC-HD kit. Four of the five African countries and three of the four South American countries received the kit/panel shipments. Two countries were unable to receive the kits and panels due to shipping challenges.

**Fig 1:**
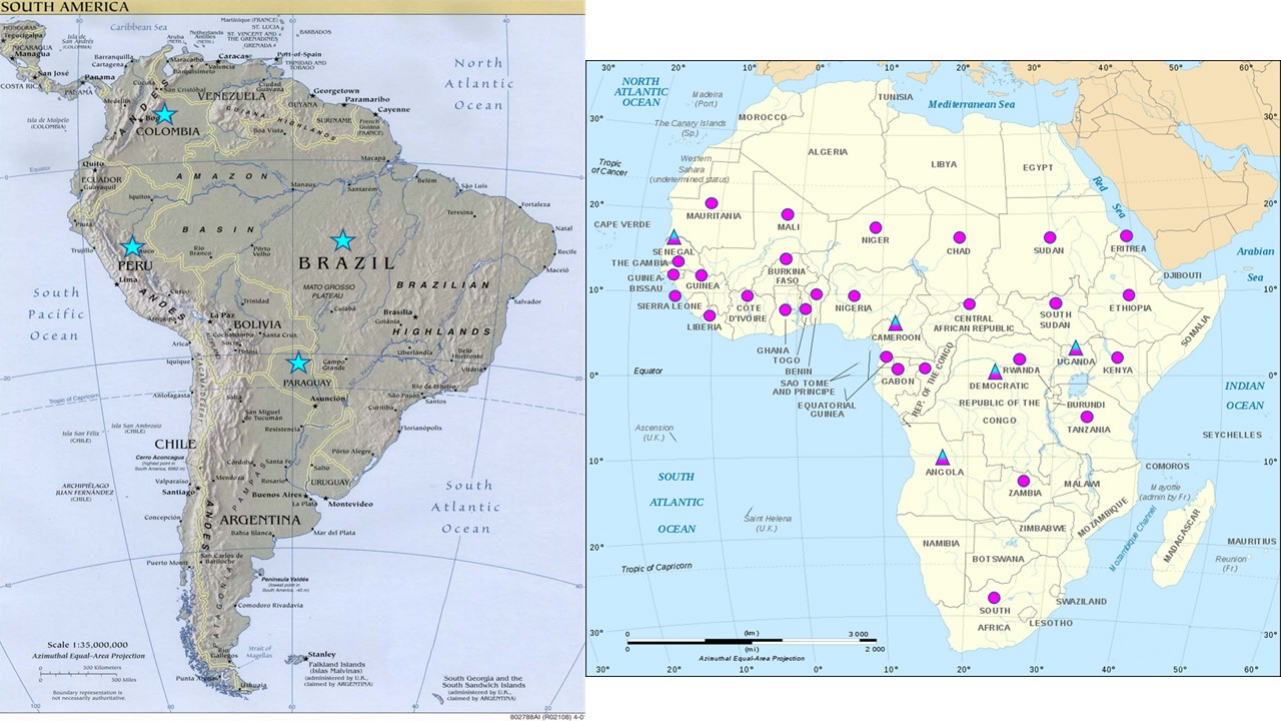
Global Map of Countries Participating in 2018 and 2019 Yellow Fever Proficiency Testing Exercises. Countries participating in 2018 YF PT only (blue star)–Brazil, Colombia, Paraguay, Peru. Countries participating in 2019 YF PT only (pink circle)–Benin, Burkina Faso, Central African Republic, Chad, Cote d’Ivoire, Equatorial Guinea, Eritrea, Ethiopia, Gabon, Ghana, Guinea, Guinea-Bissau, Kenya, Liberia, Mali, Mauritania, Niger, Nigeria, Republic of the Congo, Rwanda, Sierra Leone, South Africa, South Sudan, Sudan, Tanzania, The Gambia, Togo, Zambia. Countries participating in both 2018 and 2019 YF PT (blue/pink triangle)–Angola, Cameroon, Democratic Republic of the Congo, Senegal, Uganda. Abbreviations: YF–yellow fever; PT–proficiency testing Website link to South America map: https://www.worldofmaps.net/en/south-america/maps-of-south-america/map-of-south-america-relief-map.htm; website link to Africa map: https://www.worldofmaps.net/en/africa/africa/map-of-africa-political-map-english.htm.

In July and August 2019, two follow-up workshops were held in Africa for the purpose of YF diagnostic capacity-building, during which the YF MAC-HD kit was introduced to 33 African national laboratories. The two workshops each lasted five days and were held at the Centre Pasteur Cameroun in Yaounde, Cameroon where instruction was conducted in English, and at the Institut Pasteur de Dakar in Dakar, Senegal where instruction was conducted in French. A combined 46 laboratorians from 33 African countries at high, moderate, or potential risk of YF, attended the workshops where they trained on YF diagnostic testing methods including the YF MAC-HD kit (Fig 1). Kit instructions for use were provided in English, French, Spanish and Portuguese. At the end of the workshops, eight kits and a 10-sample YF PT panel prepared similarly to the 2018 YF PT panel were provided to each participating laboratory.

Of note, in March 2020, a third workshop was held at the Instituto de Diagnóstico y Referencia Epidemiológicos “Dr. Manuel Martínez Báez” (InDRE) in Mexico City, Mexico, for purposes of YF diagnostic capacity-building in Central and South America. This five-day workshop hosted a combined 20 laboratorians from 13 countries, and similar to the 2019 African workshops, eight kits and a 10-sample PT panel were provided to each participating laboratory at the end of the workshop. Unfortunately, due to the COVID-19 pandemic, complete YF MAC-HD PT data from all participating laboratories was unable to be obtained due to laboratories shifting their focus to SARS-CoV-2 testing. Complete YF MAC-HD PT results from this workshop will be reported at a later date.

For both 2018 and 2019 YF PT panels, participants were instructed to test the panel samples in single replicates, and if the laboratory routinely used or had access to a YF IgM in-house positive control (IHPC), the IHPC was included as an additional sample. Participants reported their results in a CDC-provided PT worksheet that captured laboratory information, kit lot, plate washing method, and sample and kit control results. The sample and control information included OD, P/N, and NBR values, along with result interpretation. A correct result was defined as performing accurate P/N and NBR calculations, along with obtaining the final correct overall interpretation (positive, negative, equivocal) for each sample. The final percentage score was calculated as the number of correct sample results obtained compared to the total number of samples tested in the PT panel. Participants were instructed to submit their results to the CDC and their respective WHO regional laboratory coordinators within three weeks after returning to their laboratory.

Additionally, the YF MAC-HD kit instructions for use included specific instructions on how to perform three plate washing methods–manual hand washing using a multichannel pipette, automated washing using a strip-well washer, or an automatic 96-well head manifold washer–in order to accommodate different plate washing methods that are used in the laboratories. YF antigen OD results from the 2019 PT exercise were analyzed according to the various plate washing methods to determine whether differences were seen in ODs when the three methods were compared.

## Results/Discussion

A summary of the YF PT exercise for both 2018 and 2019 is shown in Table 1. In 2018, of the seven countries that received kits and tested the 20-sample YF PT panel, all seven countries scored 100% on the PT. Six of these seven (86%) countries included an IHPC with the PT. In 2019, 32 of the 33 countries submitted PT results for the 10-sample YF PT panel. Of these, 31 countries scored 100% and one country scored 90% on the PT. For the one country that scored 90%, the kits and PT panel encountered a three-day delay in arrival at the home laboratory due to a flight cancellation. Fifteen of these 32 countries included an IHPC (47%). Additionally, all countries in 2018 and almost all countries (3 failed) in 2019 submitted correct results interpretations. All countries received their scores during follow-up, which included recommendations for corrective action and to assure understanding of proper YF MAC-HD results calculations as necessary.

**Table 1:** Results Summary of 2018 and 2019 Yellow Fever Proficiency Testing Exercises Performed by Laboratories in Yellow Fever Endemic Regions based on correctly identified positive or negative samples.

	2018	2019
Total countries issued YF PT	9	33
Total countries responding with PT results	7^a^	32
PT results of responding countries	7 of 7 scored 100%	31 of 32 scored 100% 1 of 32 scored 90%
Total responding countries using IHPC	6	15
Percent responding countries using IHPC	86%	47%
Countries that required follow-up due to incorrect results calculations	0	3

Abbreviations: YF–yellow fever; PT–proficiency testing; IHPC–in-house positive control

^a^Two countries were unable t receive kits due to shipping difficulties

**Fig 2:**
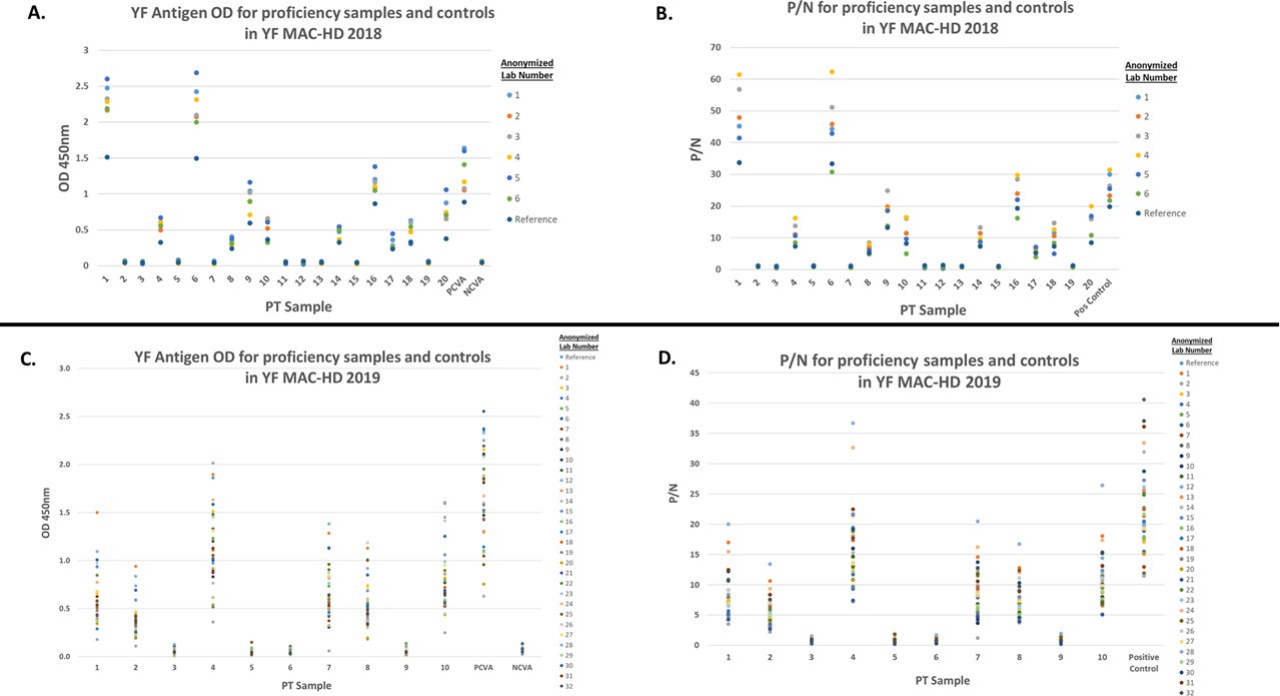
Individual Laboratory YF MAC-HD OD and P/N Values for the 2018 and 2019 Yellow Fever Proficiency Testing Exercises. A. Comparison of individual PT sample YF antigen OD values across six anonymized laboratories that participated in the 2018 YF PT Exercise. Note that the 7^th^ laboratory submitted interpreted results but did not provide OD values. B. Comparison of individual PT sample P/N values across six anonymized laboratories that participated in the 2018 YF PT Exercise. Note that the 7^th^ laboratory submitted interpreted results but did not provide P/N values. C. Comparison of individual PT sample YF antigen OD values across 32 anonymized laboratories that participated in the 2019 YF PT Exercise. D. Comparison of individual PT sample P/N values across 32 anonymized laboratories that participated in the 2019 YF PT Exercise. Abbreviations: YF–yellow fever; PT–proficiency testing; OD–optical density; P/N–defined as the mean OD of the sample reacted on YF antigen divided by the mean OD of the negative control reacted on YF antigen; PCVA–positive control reacted on YF viral antigen; NCVA–negative control reacted on YF viral antigen.

The individual laboratory OD and P/N values for both the 2018 and 2019 PT exercises were plotted to demonstrate the variability observed between the laboratories (Fig 2). As expected, generally more variability was reported in the OD and P/N values for the positive samples compared to the negative samples. Coefficient of variation (CV) values were calculated for the positive control on YF antigen (PCVA) and negative control on YF antigen (NCVA) OD values as representative samples: 2018 PCVA CV = 23.0%; 2018 NCVA CV = 21.8%; 2019 PCVA CV = 29.9%; 2019 NCVA CV = 33.2%. Even though higher-than-ideal variability was reported in OD and P/N values across laboratory results, due likely to fluctuations in local testing conditions such as high room temperature in the testing laboratory, it is important to note the interpretations for the PT samples and controls (i.e., positive, negative, equivocal) did not change and were in concordance with reference results.

**Fig 3:**
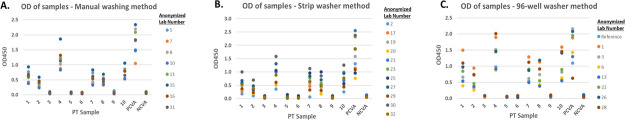
Individual Laboratory OD Values According to Three Different YF MAC-HD Washing Methods during the 2019 Yellow Fever Proficiency Testing Exercise. A. Comparison of individual PT sample YF antigen OD values across eight anonymized laboratories that performed manual hand washing using a multichannel pipette during the 2019 YF PT Exercise. B. Comparison of individual PT sample YF antigen OD values across 11 anonymized laboratories that performed automatic strip-well washing during the 2019 YF PT Exercise. C. Comparison of individual PT sample YF antigen OD values across seven anonymized laboratories that performed automatic 96-well head manifold washing during the 2019 YF PT Exercise. Abbreviations: YF–yellow fever; PT–proficiency testing; OD–optical density; PCVA–positive control reacted on YF viral antigen; NCVA–negative control reacted on YF viral antigen.

During submission of PT results, participants were requested to submit the plate washing method they used during YF MAC-HD PT testing. Of the three plate washing methods listed in the YF MAC-HD instructions for use, manual hand washing appeared to produce sample ODs with the least variation (Fig 3). CV values were again calculated for the PCVA and NCVA ODs as representative samples: manual hand washing–PCVA CV = 22.5%, NCVA CV = 26.5%; automated strip-well washing–PCVA CV = 38.4%, NCVA CV = 34.1; automatic 96-well head washing–PCVA CV = 34.6%, NCVA CV = 41.7%. The lower variability reported with manual hand washing was not entirely unexpected given the specific brand and type of automatic plate washer can vary across laboratories, leading to increased OD variation. Again, it is important to note the interpretations for the samples and controls did not change and were in concordance with reference results.

Limitations of this study include verification of whether laboratory personnel that did not attend the workshops could successfully use the YF MAC-HD kit. Also, when laboratories performed and submitted their PT results to the CDC, the participants were not required to manually calculate the results. Although some participants provided both manual and automated calculations, the ability to measure the capability of all trainees to perform manual calculations was limited. Additionally, inter-laboratory variation may have contributed to the variable OD and P/N values reported herein; for example, the participants were not required to report the room temperature of their laboratories, which may have helped indicate whether laboratory temperature indeed contributed to the higher-than-ideal OD and P/N CV values described above. Also, given laboratories within developing countries sometimes encounter challenging environments, the ability of these laboratories to comply to WHO testing performance criteria is often difficult. Lastly, the technical viability of the YF MAC-HD kit in laboratories was addressed in this manuscript; however, operational viability such as distribution and continuity of supply and costs are outside the scope of this manuscript. Nevertheless, mechanisms have recently been initiated as part of the EYE Strategy to address operational challenges to YF diagnostic testing [18].

Implementation and use of the YF MAC-HD kit is currently focused primarily on national laboratories, rather than regional/local laboratories. The use of the YF MAC-HD kit at the regional/local level, while beneficial to surveillance, would require support for infrastructure including the appropriate equipment and reliable power. These are currently available only on a limited basis. Rapid diagnostic tests for YF such as lateral flow assays may be more applicable for use in these laboratories without the burden of improving infrastructure.

The collective results from the 2018 and 2019 YF PT exercises described here demonstrate the successful transferability of the YF MAC-HD kit methods. These data also show that it was used successfully in the two continents where YF is endemic and can be used correctly under the different and sometimes challenging laboratory conditions encountered at national laboratories in these regions. Accommodation of both routine and outbreak testing is often challenging with non-standardized, in-house YF serological assays, due to the difficulty of sourcing individual reagents and reagent stock-outs. Each YF MAC-HD kit can be used to test up to 24 serum samples per plate, whereas the current in-house CDC YF MAC-ELISA accommodates only eight samples per plate. The technical viability of the YF MAC-HD kit demonstrated here lends confidence that during future outbreaks, surge capacity testing should be more easily attainable. Kit production capacity estimates indicate that projected testing volumes could be met.

Thirty-eight of 39 laboratories that submitted PT results for the YF MAC-HD kit scored 100%, one laboratory scored 90%, and approximately half of all laboratories performed the good quality control practice of using an IHPC. These data provide confidence that if the YF MAC-HD kit becomes available for routine use, it will allow laboratories in countries of high and medium YF risk to perform YF surveillance correctly and more easily than using the currently used assays. The implementation of the standardized YF MAC-HD kit will help better inform YF vaccination campaigns, leading to more efficient YF outbreak management.
